# Breakthrough Varicella During Chemotherapy in Two Vaccinated Children With Acute Leukemia

**DOI:** 10.7759/cureus.104458

**Published:** 2026-02-28

**Authors:** Hayao Kosa, Chigusa Oyama, Shigeki Nakashima, Takeshi Taketani

**Affiliations:** 1 Department of Pediatrics, Shimane University Faculty of Medicine, Izumo, JPN

**Keywords:** acyclovir, breakthrough varicella, chemotherapy, immunocompromised host, pediatric leukemia

## Abstract

Breakthrough varicella (BTV), defined as varicella occurring more than 42 days after vaccination, is typically milder than primary varicella infection. However, its clinical course in immunocompromised children remains incompletely characterized. We report two pediatric patients undergoing chemotherapy for acute leukemia who developed BTV despite prior two-dose varicella vaccination. Both patients initially presented with atypical and sparse skin eruptions, which delayed diagnosis. During chemotherapy of profound immunosuppression, the rashes rapidly progressed, accompanied by fever and neutropenia. Rapid antigen testing confirmed varicella-zoster virus infection, and early treatment with intravenous acyclovir resulted in clinical improvement without visceral complications or nosocomial transmission. These cases highlight that BTV in immunocompromised hosts might present atypically yet deteriorate quickly. Prompt recognition and early antiviral therapy are essential to prevent severe outcomes. Clinicians should consider varicella even when only a few non-vesicular lesions are observed in vaccinated children receiving chemotherapy.

## Introduction

Universal varicella vaccination has markedly reduced the incidence and severity of primary varicella infection [1]. Nevertheless, breakthrough varicella (BTV), defined as varicella occurring more than 42 days after vaccination, continues to be reported [2]. BTV is generally characterized by fewer lesions, milder symptoms, and shorter disease duration compared with primary infection [2,3]. Consequently, it may be overlooked or misdiagnosed, particularly when skin findings are subtle.

Immunocompromised children represent a special population in whom varicella infection may lead to severe or disseminated disease [4,5]. Although two-dose vaccination provides strong protection against severe outcomes, the clinical behavior of BTV during chemotherapy has not been well described. Moreover, atypical cutaneous manifestations may delay diagnosis and treatment, increasing the risk of complications and nosocomial transmission.

We report two vaccinated children receiving chemotherapy for acute leukemia who developed BTV with atypical presentations. We discuss diagnostic pitfalls and emphasize the importance of early recognition and management in immunosuppressed hosts.

## Case presentation

Case 1

A four-year-nine-month-old girl with B-cell precursor acute lymphoblastic leukemia (standard risk) [6] had completed frontline therapy at four years of age. Nine months later, she was diagnosed with late combined bone marrow and extramedullary relapse with central nervous system involvement and hydrocephalus. Reinduction chemotherapy was initiated after a 14-day course of prednisolone and consisted of intravenous high-dose methotrexate (MTX), vincristine, L-asparaginase, and dexamethasone, along with intrathecal (IT) methotrexate (MTX), cytarabine (Ara-C), and hydrocortisone (HDC).

She had received two doses of the varicella vaccine and demonstrated positive pre-treatment antibody titers (5.2; reference range: 2.0). At admission, her temperature was 38.2°C, pulse was 123/min, blood pressure was 102/68 mmHg, and oxygen saturation was 98% on room air. Cardiopulmonary and abdominal examinations were unremarkable. Several small papules on the face were initially diagnosed as molluscum contagiosum.

During induction therapy, she developed febrile neutropenia (FN) (Table 1), and broad-spectrum antibiotics were administered without granulocyte colony-stimulating factor (G-CSF). The blood culture analysis revealed the presence of methicillin-resistant coagulase-negative staphylococcus (MRCNS).

**Table 1:** Laboratory findings in febrile neutropenia WBC: White blood cell; T. bil: Total bilirubin; AST: Aspartate aminotransferase; ALT: Alanine aminotransferase; LDH: Lactate dehydrogenase; GGT: Gamma-glutamyl transferase; BUN: Blood urea nitrogen; CRP: C-reactive protein; MRCNS: Methicillin-resistant coagulase-negative staphylococcus; VZV-IgG: Varicella-zoster virus immunoglobulin G; VZV-IgG was measured using an enzyme immunoassay (EIA).

Parameters	Value of Case 1	Value of Case 2	Unit	Reference Range
Complete blood count				
WBC	350	480	/μL	5,000–14,500
Blast	0	0	%	
Neutrophil	0	1.7	%	
Lymphocyte	100	98.3	%	
Hemoglobin	9.6	7.3	g/dL	11.5–13.5
Platelet	2.6 × 10^3^	3.0 × 10^3^	/μL	150–350 × 10^3^
Blood chemistry				
Total protein	6.0	6.2	g/dL	6.5-8.0
Albumin	2.9	3.9	g/dL	3.6-5.0
T. bil	2.8	0.9	mg/dL	0.2-1.2
AST	44	10	U/L	10-40
ALT	325	21	U/L	5-40
LDH	144	172	U/L	120-240
GGT	142	16	U/L	9-132
BUN	16.3	9.6	mg/dL	8-20
Creatinine	0.22	0.42	mg/dL	0.4-1.1
CRP	26.16	0.9	mg/dL	<0.2
Blood culture	MRCNS	Negative		Negative
VZV-IgG index	5.2	8.1		<2.0

**Figure 1:**
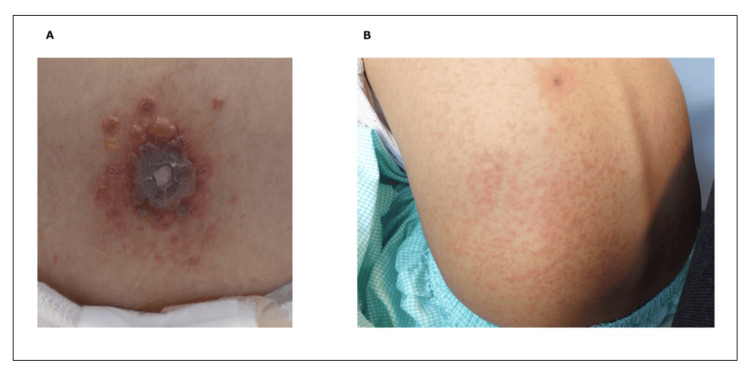
Cutaneous manifestations of breakthrough varicella in two immunocompromised children undergoing chemotherapy (A) Case 1: Multiple erosive and vesicular lesions on the trunk and extremities, rapidly developing from initially sparse papules. (B) Case 2: Generalized erythematous and vesicular eruptions on the trunk and extremities.

Subsequently, the skin lesions increased rapidly in number and evolved into erosive and vesicular eruptions involving the trunk, extremities, and palms (Figure 1, Panel A). Dermatological consultation raised suspicion for varicella. A rapid antigen test for varicella-zoster virus (VZV) was positive, and intravenous acyclovir (ACV) was immediately started. The lesions gradually crusted over, and the patient recovered without complications. No nosocomial infections occurred.

Case 2

A 6-year-10-month-old girl was referred for recurrent fever and pancytopenia and was diagnosed with acute myeloid leukemia classified as M2 according to the French-American-British (FAB) classification [7]. Induction chemotherapy was initiated (intravenous administration: etoposide, Ara-C, and mitoxantrone; IT injection: MTX, Ara-C, and HDC). She had previously ​​​​​received two doses of the varicella vaccine, with positive antibody titers (8.1; reference range: 2.0). At admission, her vital signs were stable, and no rash was noted. During induction therapy, she developed FN (Table 1) and was treated with antibiotics and antifungal agents, without the use of G-CSF. A few small, dome-shaped vesicles appeared on the trunk and extremities. Initially mild and sparse, the eruptions rapidly progressed to generalized erythema with vesicular lesions (Figure 1, Panel B).

Rapid antigen testing confirmed VZV infection. Intravenous ACV was administered promptly. However, the fever persisted, and the rash continued to spread throughout the body even after ACV administration, prompting the addition of intravenous immunoglobulin as an adjunctive therapy. After that, the lesions resolved with crust formation, and no secondary transmission occurred.

## Discussion

This report describes two vaccinated children who developed BTV during intensive chemotherapy. BTV is generally considered milder than primary varicella in immunocompetent individuals, typically presenting with fewer lesions and less systemic involvement [2,3]. However, the clinical course in immunocompromised patients is less clearly characterized. Our cases provide additional clinical insights into how BTV might present and evolve under profound immunosuppression.

In both patients, the initial cutaneous manifestations were sparse and atypical, leading to diagnostic uncertainty. This is consistent with the known clinical characteristics of BTV in vaccinated individuals, which often include fewer lesions and milder symptoms compared with primary infection [2,3]. Importantly, however, once chemotherapy-induced immunosuppression progressed, the skin eruptions increased rapidly and became more generalized. Previous studies have reported that, although vaccination reduces disease severity overall, immunocompromised patients remain at risk for severe complications and even fatal outcomes [4,5]. Taken together, these observations suggested that BTV in immunocompromised hosts might initially resemble the mild phenotype observed in immunocompetent vaccinated individuals while still retaining the potential for rapid progression and clinical deterioration.

These features highlight the importance of early recognition. As the initial presentation is subtle and nonspecific, clinical diagnosis alone can be difficult [2,3]. Laboratory confirmation, therefore, plays a critical role. PCR-based methods are considered highly sensitive for confirming VZV infection, particularly in atypical cases [2]. However, genetic testing is not always immediately available in routine clinical settings. In our cases, rapid antigen testing facilitated prompt diagnosis and enabled early therapeutic intervention. This supports the practical value of accessible diagnostic tools, such as PCR or antigen-based assays, for early detection in immunocompromised patients with minimal or atypical skin findings.

Early diagnosis also has important implications for infection control. Varicella is highly transmissible, and outbreaks in immunocompromised hospital populations have been reported [8,9]. In both of our patients, early recognition of possible varicella led to timely diagnostic confirmation, isolation, and implementation of infection control measures. No secondary transmission occurred. Although causal inference cannot be established from two cases, these observations demonstrated that prompt identification of varicella contributes to the prevention of nosocomial spread in high-risk settings.

Early antiviral therapy represents another key component of management. Intravenous acyclovir has been shown to reduce viral dissemination and prevent severe complications in immunocompromised children with varicella [10-12]. In our cases, antiviral treatment was initiated promptly after diagnostic confirmation, and neither patient developed visceral involvement such as severe pneumonia, hepatitis, or encephalitis. While the clinical course cannot be attributed solely to treatment in an observational report, these findings were consistent with previous evidence suggesting that early acyclovir administration may help limit disease progression as well as reduce the risk of complications in immunocompromised hosts.

Another clinically relevant observation is that both patients developed BTV despite prior two-dose vaccination and detectable antibody titers. Similar cases have been reported in pediatric oncology patients, indicating that vaccine-induced immunity might be insufficient to fully prevent infection during periods of profound immunosuppression [13,14]. Under the current Japanese immunization program, routine two-dose varicella vaccination has been implemented since 2014, with doses administered at 12-15 months of age and at least three months later. Although two-dose vaccination generally induces high initial seroconversion rates, longitudinal studies have demonstrated that both antibody positivity and geometric mean concentrations decline over time after vaccination [3,15,16], and breakthrough infection may occur as humoral immunity wanes. Moreover, protection against varicella relies substantially on cell-mediated immunity, which may be profoundly impaired during intensive chemotherapy. Importantly, there have been documented cases in immunocompromised patients where the varicella vaccine strain has been known to reactivate, resulting in severe varicella [17,18], although we could not determine whether the cause of our breakthrough varicella cases was the wild-type strain or the vaccine strain. These findings suggest that vaccination history or seropositivity alone may not reliably predict protection in immunocompromised children undergoing chemotherapy and that the possibility of varicella should not be excluded when new skin lesions appear during treatment.

Taken together, our cases illustrate a clinically important pattern: BTV in immunocompromised children may initially present with limited and atypical skin findings, similar to those seen in immunocompetent vaccinated individuals, but can subsequently progress rapidly under conditions of impaired cellular immunity. In this context, maintaining a high index of suspicion, performing early diagnostic testing using PCR or antigen-based methods, initiating prompt antiviral therapy, and implementing appropriate isolation measures may be important strategies to reduce the risk of severe disease and nosocomial transmission.

While limited by the small number of cases and the absence of virological characterization, this report reinforces practical lessons for clinicians caring for immunocompromised pediatric patients.

## Conclusions

BTV can occur in fully vaccinated children undergoing chemotherapy and may initially present with subtle, atypical skin findings. However, rapid progression is possible during immunosuppression. Early suspicion, prompt diagnostic testing, and immediate antiviral therapy are critical to prevent severe complications and nosocomial transmission. Clinicians should consider varicella in any new rash developing in immunocompromised pediatric patients, regardless of vaccination history.
